# Prevalence of Nutrition Standards Use by Municipalities in Government-Owned or Operated Properties, United States, 2021

**DOI:** 10.3390/nu18071165

**Published:** 2026-04-07

**Authors:** Reena Oza-Frank, Amy Lowry Warnock, Diane M. Harris

**Affiliations:** Division of Nutrition, Physical Activity, and Obesity, National Center for Chronic Disease Prevention and Health Promotion, Centers for Disease Control and Prevention, Atlanta, GA 30341, USA; hwf9@cdc.gov (A.L.W.); hva6@cdc.gov (D.M.H.)

**Keywords:** food service guidelines, local government, healthy food access, policy, public health nutrition

## Abstract

**Background/Objectives:** Adopting written nutrition standards for food sold or served by local governments is a strategy for increasing access to healthier options among employees and residents. **Methods:** We used data from a 2021 national survey of 1982 municipal governments serving populations of 1000 or more. Among municipalities that sell or serve food or beverages, we examined the prevalence and 95% confidence intervals (CI) of those with written nutrition standards. Logistic regression models were used to obtain odds ratios and 95% CIs of written nutrition standards by municipality characteristics. Finally, we examined the prevalence including nutrition standards in food purchasing agreements or food service contracts among municipalities that sell or serve food and have written nutrition standards. **Results:** Among U.S. municipalities in 2021, 32% reported selling and 21% reported serving food or beverages. Among U.S. municipalities that sell or serve food or beverages, the prevalence of municipalities with written nutrition standards was 19%, and of these, 78% reported including their written nutrition standards in city food purchasing agreements or food service contracts. In adjusted analyses, the region (West vs. Midwest adjusted odds ratio [aOR]: 2.9 [95% CI: 1.7, 4.9]) and presence of a food policy council remained significantly associated with having written nutrition standards (aOR: 1.7 [1.1, 2.5]). **Conclusions:** Although only 1 in 5 municipalities that sell or serve food or beverages have written nutrition standards, of those that do, almost 80% reported including the standards in contracts, highlighting an important implementation lever and a public health opportunity for communities to adopt standards that offer healthy food and beverage options in public spaces.

## 1. Introduction

Three in four US adults report at least one chronic condition (e.g., heart disease, cancer, diabetes, obesity, hypertension) [1]. Diets high in sugar, sodium, and saturated fat and low in whole grains, fruits, vegetables, and fiber are associated with developing chronic conditions [2]. Improving food environments by providing healthy foods and beverages where people work, study, and live helps make it easier to make healthy food choices [3].

To improve food environments, federal and local governments have been working towards implementing nutrition standards where food and beverages are served and sold to increase the availability of healthy offerings [4,5,6,7]. For example, the Food Service Guidelines (FSG) for Federal Facilities is intended for use in government worksites and other community institutions (e.g., public hospitals, parks and recreation centers, colleges and universities, etc.) where food is served or sold. Adopting FSG can increase access to healthy food offerings, while simultaneously supporting population nutrition and health goals [8,9]. Specifically, FSG includes nutrition standards aligned with the Dietary Guidelines for Americans [2] to increase access to fruits, vegetables, and whole grain products, and to reduce added sugars. FSG were developed by an interagency working group consisting of nine federal agencies.

As of 2024, local governments employed over 14 million people in the U.S. [10], exceeding federal and state governments in the number of job gains in 2023 [11]. As such, local governments have the potential to support healthy eating by impacting the significant numbers of government employees through the foods served in building cafeterias and vending machines. Local governments can also support healthy eating among other city residents. For example, local agencies procure food for use in public hospitals, afterschool programs, senior centers, and correctional facilities. A recent simulation study showed that the implementation of FSG nutrition standards in federal government and private worksites would result in significant lifetime healthcare costs savings ($212 million) because of averted cases of diet-related cardiovascular disease and mortality [12]. Thus, public health authorities have recognized the adoption of nutrition standards in local, state, and federal food service and procurement contracts as a lever for improving population health outcomes through increasing demand for healthy food offerings [13,14,15,16].

However, as of 2016, only 5 of the 20 most populous cities in the U.S. had adopted a policy with written nutrition standards [7]. Furthermore, the 2014 National Survey of Community-Based Policy and Environmental Supports for Healthy Eating and Active Living (CBS-HEAL) conducted by the Centers for Disease Control and Prevention (CDC) found that only 3.2% of U.S. municipalities reported written nutrition standards, with greater prevalence observed among large municipalities [17].

The prevalence of selling or serving foods and beverages within municipalities remains unknown, as is the prevalence of formal written nutrition standards and examining how those standards relate to municipal characteristics and contractual implementation tools. To assess more recent uptake of nutrition standards in municipal-level policies and practices, the present study investigates 1982 municipalities surveyed in the 2021 CBS-HEAL survey. Our study objectives were to assess: (1) the prevalence of U.S. municipalities that sell or serve food or beverages to employees or visitors on local government-owned or operated properties; (2) the prevalence of municipalities with written nutrition standards and prevalence differences by characteristics among municipalities that sell or serve food or beverages; and (3) the prevalence of incorporating nutrition standards into contracts or purchasing agreements among U.S. municipalities that reported having written nutrition standards for selling or serving food or beverages.

## 2. Materials and Methods

### 2.1. Survey and Study Sample

The 2021 CBS-HEAL survey is a nationally representative survey of U.S. municipalities with 1000 or more residents. Methods for the 2021 survey have been published [18]. Briefly, survey data was collected from May 2021 through September 2021. The sampled municipalities were drawn from the 2017 U.S. Census of Governments [19], which was the most recent data available at the time of survey. The survey used explicitly stratified sampling by U.S. Census region (Northeast, Midwest, South, and West) and urban/rural status, which was defined on the basis of the proportion of a Census place’s population that resides within a Census-designated urban area. Further implicit stratification, performed by sorting by population size, was also used in each stratum to ensure that small, medium, and large municipalities from each stratum were included in the sample. The survey was sent to the city or town manager, city planner, city administrator or a similar role in each municipality to complete the survey electronically (>80% of respondents) or by paper or interviewer-administered telephone survey (by request). In 2021, 4417 municipalities were sampled, and 1982 completed the survey (45% response rate; final analytic sample size). More information about the survey methodology can be found at https://stacks.cdc.gov/view/cdc/131806 (accessed on 5 March 2026).

### 2.2. Measures

To assess the prevalence of municipalities that sell or serve food or beverages to employees or visitors on local government-owned or operated properties, respondents answered two separate questions. Respondents were asked, “Not including schools, does your local government or a subcontractor sell foods or beverages to employees or visitors on local government-owned or operated properties? *This could include cafeterias, vending machines, park concession stands, or other food venues*.” and “Not including schools, does your local government or a subcontractor serve food (at little or no cost) to facility residents or program participants in facilities or programs owned or operated by the local government? *This could include correctional facilities, senior centers/programs, recreation programs, or other settings that serve congregate meals.*” Similarities by demographics and small sample sizes for some demographics for those serving foods or beverages led to the decision to combine municipalities that serve or sell foods or beverages. Respondents who answered “Yes” to either question for selling or serving food or beverages were considered affirmative. Respondents answering “No” were considered as not selling or serving food or beverages to employees or visitors on local government-owned or operated properties. For this study, municipalities with missing or “Do not know” responses on selling (*n* = 46) or serving (*n* = 104) foods or beverages to employees or visitors on local government-owned or operated properties were excluded from the analyses involving those survey questions.

If respondents answered “Yes” to either question for selling or serving food or beverages, follow-up questions included, “Not including schools, does your local government have written nutrition standards for foods and beverages sold to employees or visitors in or on government-owned or operated properties?” and “Not including schools, does your local government have written nutrition standards for foods served to facility residents or program participants in facilities or programs it owns or operates?” (respectively). Finally, if respondents answered “Yes” to having written nutrition standards for serving or selling food or beverages, the following question was asked after each affirmative response: “Are any of these nutrition standards included in food purchasing agreements and/or food service contracts?”.

### 2.3. Statistical Analysis

All analyses were conducted by using survey procedures in SAS version 9.4 (SAS Institute Inc., Cary, NC, USA) to account for design variables, nonresponse, and sample weights. Municipal characteristics were derived from the 2020 American Community Survey 5-year estimates [20]. Characteristics included population size (1000–2499, 2500–49,999, or ≥50,000), rural/urban status (based on whether ≥50% of population for a municipality reside in an urban area), U.S. Census region (Northeast, Midwest, South, or West) [21], median educational attainment (≥some college or ≤high school graduate), percentage of the population living below the Federal Poverty Level (<20% or ≥20%) to reflect persistent poverty as defined by the U.S. Department of Agriculture [22], racial/ethnic composition of the municipality (>90% non-Hispanic White, 51% to 89% non-Hispanic White, or ≤50% non-Hispanic White), and whether or not the community has a “local, county, or regional food policy council, food security coalition, or other community group working to increase access to healthy food. (*A food policy council is a council that brings together stakeholders from diverse food-related sectors in a specific geographical area to examine how the food system is operating in that area and to develop recommendations for improvement.*)”.

We examined the prevalence of municipalities that sell or serve food or beverages to employees or visitors on local government-owned or operated properties overall and by municipality characteristics. Among municipalities that sell or serve food or beverages, we examined the prevalence and 95% confidence intervals (CI) of those with written nutrition standards. We used chi-square tests to determine differences in the prevalence of written nutrition standards by municipality characteristics with *p* < 0.05 set as significance. Reliability of estimates and collinearity were assessed to verify the stability of included variables. Logistic regression models were used to obtain odds ratios and 95% CIs of written nutrition standards, adjusted for all municipality characteristics. Finally, we examined the prevalence of municipalities that sell or serve food (combined) with written nutrition standards included in food purchasing agreements or food service contracts.

The Centers for Disease Control and Prevention (CDC) and the National Opinion Research Center (NORC) at the University of Chicago both determined the survey was not human subjects research and did not need IRB review.

## 3. Results

Nonresponse bias analysis was conducted after data collection was complete, and although there was variation in responses by region and urbanicity, these characteristics were part of the sample design; responses did not vary significantly by other characteristics. Most municipalities in this sample had a population of 2500 to 49,999 people (weighted frequency: 58.6%), were urban (75.5%), were in the South (36.2%) or Midwest (35.0%), had <20% poverty prevalence (78.7%), had a population median education level of some college or higher (67.7%), had a racial/ethnic distribution of 51% to 89% non-Hispanic White (53.8%), and did not have a food policy council (72.4%) (Table 1). Among U.S. municipalities in 2021, 39.7% of municipalities reported selling or serving food or beverages (combined), (i.e., 31.7% reported selling and 21.3% reported serving food or beverages); municipality characteristics by selling or serving food or beverages followed similar patterns (Table 1).

**Figure 1:**
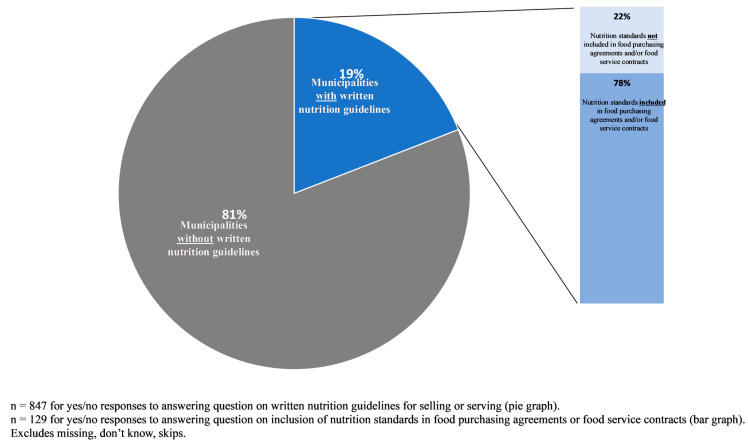
Percent of municipalities that sell or serve foods or beverages to employees or visitors on local government-owned or operated properties with written nutrition guidelines and nutrition standards included in food purchasing agreements or food service contracts.

Among U.S. municipalities that sell or serve food or beverages, the prevalence of municipalities with written nutrition standards was 19.1% (Table 2, Figure 1), and of these, 77.6% reported including their written nutrition standards in city food purchasing agreements or food service contracts (Figure 1). In adjusted analyses, region (West vs. Midwest adjusted odds ratio [aOR]: 2.9 (95% CI: [1.7, 4.9]), racial/ethnic composition (≤50% non-Hispanic White vs. >90% non-Hispanic White; aOR: 2.3 [1.1, 4.8]), and presence of a food policy council (aOR: 1.7 [1.1, 2.5]) remained significantly associated with having written nutrition standards.

## 4. Discussion

In 2021, just 1 in 5 municipalities that sell or serve food or beverages to employees or visitors on local government-owned or operated properties reported having written nutrition standards, which is an important public health strategy for defining the types of healthy meals, snacks, and beverages that are served or sold in these facilities. Among municipalities with written nutrition standards, over three-quarters reported including them in food purchasing agreements or food service contracts, illustrating an implementation lever local officials can use to formalize and operationalize written nutrition standards to improve diet quality and create healthier food environments.

The current study found that the use of written nutrition standards was greater among municipalities located in the Western U.S. This finding is consistent with the previous CBS survey (2014) [7], where 3 of 6 identified nutrition policies were more likely to be adopted on the West coast [7]. Another compilation of local public health policies hosted by a nonprofit organization summarizes that as of 2024, 27 of the top 75 most populous U.S. cities have a healthy food procurement policy [23]. This study also notes higher prevalence of nutrition standards and healthy food procurement policies in the Western U.S., which is consistent with the results of this study. Municipalities in the West or in other regions of the U.S. interested in adopting written nutrition policies can use policies identified in these studies as models for their own efforts.

Although this study was cross-sectional, limiting causal inference, the positive association between having a food policy council and the presence of written nutrition standards observed is supported by previous studies. Specifically, previous research indicates a positive association between the presence of a food policy council and the existence of health-promoting policies that support population goals for reducing chronic disease [24,25]. Municipalities with food policy councils may differ from others in political commitment, institutional capacity, funding structures, local advocacy, or pre-existing health priorities. A better understanding of “if” and “how” food policy councils influence the development of local nutrition standards may help guide local municipalities. Additionally, implementing aspects of a food policy council, including capacity, funding, and resource allocation, that have been previously published [26,27] could provide valuable information for local government officials looking to use their existing councils to support the adoption of written nutrition standards, or information on how to establish such councils in their own jurisdictions.

The reason(s) for the low prevalence of written nutrition standards in municipalities are not known, and implementation research may help to understand the capacity of municipal governments to formulate, adopt, and implement nutrition standards. Such data may help identify best practices, successful strategies, and a “roadmap” for wider dissemination and adoption. A recent study in Los Angeles County found that constraints to FSG implementation included factors related to staffing, training, and infrastructure [28]. However, a key finding from this study was that having a written nutrition policy can facilitate standardizing the inclusion of nutrition standards in contracts [28], supporting the results of our study.

Another reason for the low prevalence of written nutrition standards may be the lack of incentives for local officials to adopt nutrition standards. True cost studies, or studies that assess how the costs and benefits of FSG implementation in government facilities may affect people, both directly and indirectly, would help support the business case for adopting written nutrition standards. Local health officials could use such data to educate officials in municipal departments on the costs, to guide decisions about adopting nutrition standards as an opportunity to improve healthy food offerings with the goal of improving the health of their citizens.

Policy adoption is an important first step, but essential next steps such as policy implementation (e.g., fidelity, compliance, monitoring) and evaluating policy effectiveness are important to understand the policy impact on changes in food purchasing and consumption patterns. Evaluations of FSG implementation have shown improvements to the food environment through increased availability of healthier foods and beverages in government facilities [29,30], and increased cafeteria and vending purchases of healthier foods in schools, hospitals, and other settings [31,32,33,34]. For example, the use of pricing incentives increases sales of healthier food and drink selections in worksites, parks, and other venues [35]. Additional evaluations may provide further rationale and support for local governments interested in adopting written nutrition standards.

### Limitations

The findings from this study are subject to some limitations. Survey data were self-reported information from city manager planners and similar staff, which may have resulted in misclassification because we could not confirm the selling or serving of food or beverages or the presence of written nutrition standards. Nonresponse bias analysis was conducted and the analysis showed minimal impact on results, however there may have been unmeasured external influences that could have affected participation rates (e.g., role of individual responding to the survey). Although the survey’s findings may not be generalizable to very small municipalities (≤1000 people), the survey pilot revealed that these municipalities only accounted for 3% of the U.S. municipal population [36]. Despite being administered from May through to September 2021, the survey was not written to capture the COVID-19 pandemic context or potential pandemic impacts on the existence of nutrition standards within municipalities. The analysis combined municipalities that sell or serve food, limiting the ability to identify varying policy contexts and/or implementation challenges. Finally, the survey did not ask about policy content [37] (e.g., what nutrition standards were included or venues the standards covered [e.g., cafeterias, vending machines]); thus, we could not assess the comprehensiveness of the policies, and varying degrees of policies (stronger vs. weaker) were both counted as a policy in this study. Despite the limitations, the study is nationally representative, and the data presented are based on unique questions not asked in other national surveys.

## 5. Conclusions

In conclusion, although only 1 in 5 municipalities that sell or serve food or beverages have written nutrition standards, of those that do, almost 4 in 5 reported including the standards in contracts, highlighting an important implementation lever and a public health opportunity for communities to adopt nutrition standards to offer healthy food and beverage options in public spaces and improve food environments under the jurisdiction of municipal governments. Implementation research on how nutrition standards are developed, adopted, operationalized, and evaluated would provide additional understanding of nutrition policy across different municipal contexts.

## Figures and Tables

**Table 1:** Characteristics of U.S. Municipalities with Population Size ≥1000 People Overall and by Municipalities That Sell or Serve Food or Beverages to Employees or Visitors on Local Government-Owned or Operated Properties, National Community Based Survey of Supports for Healthy Eating and Active Living, 2021.

Municipal Characteristics	All Municipalities (*n* = 1982) ^c^ (%) ^d^	Municipalities That Sell Food or Beverages to Employees or Visitors on Local Government-Owned or Operated Properties (*n* = 1936) ^a^ (%) ^d^	Municipalities That Serve Food or Beverages to Employees or Visitors on Local Government-Owned or Operated Properties (*n* = 1878) ^b^ (%) ^d^
All municipalities	100	31.7	21.3
Population size			
1000 to <2500 people	33.9	5.5	4.3
2500 to 49,999 people	58.6	20.6	13.2
≥50,000 people	7.5	5.6	3.7
Rural/urban status			
Urban	75.5	26.9	17.4
Rural	24.5	4.8	3.8
Census region			
Northeast	14.2	3.4	2.7
Midwest	35.0	11.5	5.6
South	36.2	11.6	8.0
West	14.6	5.3	4.9
Poverty prevalence			
<20%	78.7	25.2	15.8
≥20%	21.3	6.5	5.4
Median educational attainment			
≤High school graduate	32.3	8.7	7.0
≥Some college	67.7	23.0	14.2
Racial/ethnic composition			
≥90% Non-Hispanic White	29.4	7.8	3.4
51% to 89% Non-Hispanic White	53.8	18.3	12.4
≤50% Non-Hispanic White	16.8	5.5	5.5
Presence of food policy council working to increase healthy food access			
Yes	27.6	10.6	8.5
No	72.4	21.1	12.8

^a^ Excludes *n* = 46 municipalities with missing or do not know responses to the survey question, “Not including schools, does your local government or a subcontractor sell foods or beverages to employees or visitors on local government-owned or operated properties? *This could include cafeterias, vending machines, park concession stands, or other food venues*.”. ^b^ Excludes *n* = 104 with missing or do not know responses to the survey question, “Not including schools, does your local government or a subcontractor serve food (at little or no cost) to facility residents or program participants in facilities or programs owned or operated by the local government? *This could include correctional facilities, senior centers/programs, recreation programs, or other settings that serve congregate meals.*” ^c^ Unweighted sample sizes are presented. ^d^ Weighted percentages are presented. Due to rounding, weighted percentage may not add up to 100%.

**Table 2:** Prevalence of and Adjusted Odds Ratios of Written Nutrition Standards Among U.S. Municipalities that Sell or Serve Food or Beverages to Employees or Visitors on Local Government-Owned or Operated Properties by Municipality Characteristics, National Community Based Survey of Supports for Healthy Eating and Active Living, 2021.

Municipal Characteristics	% (95% CI) ^b^	*p*-Value ^c^	Adjusted Odds Ratio ^d^ (95% CI)
Municipalities that Sell or Serve ^a^ Food or Beverages to Employees or Visitors on Local Government-Owned or Operated Properties	19.1 (16.4, 21.7)	n/a	n/a
Population size (*n* = 847) ^e^			
1000 to <2500 people (*n* = 183)	19.9 (13.8, 26.0)	<0.0001	Reference
2500 to 49,999 people (*n* = 531)	15.2 (12.2, 18.4)		0.7 (0.3, 1.5)
≥50,000 people (*n* = 133)	35.1 (27.0, 43.3)		1.6 (0.7, 3.7)
Rural/urban status (*n* = 842)			
Urban (*n* = 685)	18.7 (15.7, 21.6)	0.4	Reference
Rural (*n* = 157)	21.7 (14.9, 28.6)		1.5 (0.7, 3.2)
Census region (*n* = 847)			
Northeast (*n* = 97)	19.7 (11.6, 27.8)	<0.0001	1.8 (0.9, 3.5)
Midwest (*n* = 284)	11.1 (7.5, 14.6)		Reference
South (*n* = 245)	21.1 (15.1, 25.2)		1.5 (0.9, 2.6)
West (*n* = 221)	32.7 (26.4, 39.1)		2.9 (1.7, 4.9)
Poverty prevalence (*n* = 847)			
<20% (*n* = 667)	17.5 (14.6, 20.3)	0.03	Reference
≥20% (*n* = 180)	24.6 (18.2, 31.0)		1.2 (0.8, 2.0)
Median educational attainment (*n* = 847)			
≤High school graduate (*n* = 242)	21.8 (16.5, 27.1)	0.2	1.3 (0.8, 2.1)
≥Some college (*n* = 605)	17.9 (14.8, 20.9)		Reference
Racial/ethnic composition (*n* = 847)			
≥90% Non-Hispanic White (*n* = 205)	10.4 (6.2, 14.6)	<0.0001	Reference
51% to 89% Non-Hispanic White (*n* = 478)	19.2 (15.6, 22.8)		1.8 (1.0, 3.3)
≤50% Non-Hispanic White (*n* = 164)	29.7 (22.8, 36.7)		2.3 (1.1, 4.8)
Food policy council working to increase healthy food access (*n* = 844)			
Yes (*n* = 274)	26.2 (21.0, 31.5)	0.0002	1.7 (1.1, 2.5)
No (*n* = 570)	15.4 (12.4, 18.4)		Reference

Abbreviations: CI, confidence interval, ^a^ *n* = 847 due to survey skip patterns. ^b^ Weighted percentage are presented. Due to rounding, weighted percentage may not add up to 100%. ^c^ χ^2^ test was used for each variable to examine differences across categories, and *p* value < 0.05 was considered statistically significant. ^d^ All municipal characteristics were included in the model. ^e^ Unweighted sample sizes are presented.

## Data Availability

The data presented in this study are openly available in https://data.cdc.gov/Nutrition-Physical-Activity-and-Obesity/National-Community-Based-Survey-of-Supports-for-He/8hus-y5nc/about_data (accessed on 5 March 2026).

## References

[1] Watson K.B., Wiltz J.L., Nhim K., Kaufmann R.B., Thomas C.W., Greenlund K.J. (2025). Trends in Multiple Chronic Conditions Among US Adults, By Life Stage, Behavioral Risk Factor Surveillance System, 2013–2023. Prev. Chronic Dis..

[2] U.S. Department of Agriculture, U.S. Department of Health and Human Services Dietary Guidelines for Americans, 2025–2030. https://cdn.realfood.gov/DGA.pdf.

[3] Swinburn B.A., Sacks G., Hall K.D., McPherson K., Finegood D.T., Moodie M.L., Gortmaker S.L. (2011). The global obesity pandemic: Shaped by global drivers and local environments. Lancet.

[4] Auvinen A., Marcinkevage J., Mornick C., Nambuthiri S., Daniel M., Carney B., Prather C., Dolan J. (2021). Improving the food environment in Washington state-run correctional facilities: The Healthy Commissary Project. Am. J. Public Health.

[5] Kimmons J., Wood M., Villarante J.C., Lederer A. (2012). Adopting healthy and sustainable food service guidelines: Emerging evidence from implementation at the United States Federal Government, New York City, Los Angeles County, and Kaiser Permanente. Adv. Nutr..

[6] Lowry-Warnock A., Strombom N., Mugavero K., Harris D., Blanck H.M., Onufrak S. (2023). Advancing healthy food service in the United States: State food service guidelines policy adoption and implementation supports, 2015–2019. Am. J. Health Promot..

[7] Zaganjor H., Bishop Kendrick K., Onufrak S., Ralston Aoki J., Whitsel L.P., Kimmons J. (2019). Food service guideline policies on local government-controlled properties. Am. J. Health Promot..

[8] Niebylski M.L., Lu T., Campbell N.R., Arcand J., Schermel A., Hua D., Yeates K.E., Tobe S.W., Twohig P.A., L’Abbé M.R. (2014). Healthy food procurement policies and their impact. Int. J. Environ. Res. Public Health.

[9] Food Service Guidelines Federal Workgroup. Food Service Guidelines for Federal Facilities. U.S. Department of Health and Human Services, 2017. https://www.cdc.gov/obesity/downloads/guidelines_for_federal_concessions_and_vending_operations.pdf.

[10] Ennis K., Jacobson R., Maloney D., Saxon N., Segal J., Wenning J., Wilburn S. Annual Survey of Public Employment & Payroll Summary Report: 2024. 27 March 2025. Report No.: Contract No.: G24-ASPEP. https://www.census.gov/content/dam/Census/library/publications/2024/econ/ASPEP%20Summary%20Report%202024.pdf.

[11] U.S. Bureau of Labor Statistics Employment in Government Rose by 709,000 in 2023. The Economics Daily. https://www.bls.gov/opub/ted/2024/employment-in-government-rose-by-709000-in-2023.htm.

[12] Abrahams-Gessel S., Wilde P., Zhang F.F., Lizewski L., Sy S., Liu J., Ruan M., Lee Y., Mozaffarian D., Micha R. (2022). Implementing federal food service guidelines in federal and private worksite cafeterias in the United States leads to improved health outcomes and is cost saving. J. Public Health Policy.

[13] Glickman D., Parker L., Sim L.J., Del Valle Cook H., Miller E.S. (2012). Accelerating Progress in Obesity Prevention: Solving the Weight of the Nation.

[14] Khan L.K., Sobush K., Keener D., Goodman K., Lowry A., Kakietek J., Zaro S. (2009). Recommended community strategies and measurements to prevent obesity in the United States. MMWR Recomm. Rep..

[15] World Health Organization (2021). Action Framework for Developing and Implementing Public Food Procurement and Service Policies for a Healthy Diet. https://www.who.int/publications/i/item/9789240018341.

[16] Parker L., Burns A.C., Sanchez E. (2009). Local Government Actions to Prevent Childhood Obesity.

[17] Onufrak S.J., Zaganjor H., Moore L.V., Carlson S., Kimmons J., Galuska D. (2016). Nutrition standards for food service guidelines for foods served or sold in municipal government buildings or worksites, United States, 2014. Prev. Chronic Dis..

[18] Onufrak S.J., Moore L.V., Pierce S.L., MacGowan C.A., Galuska D.A. (2023). Changes in policy supports for healthy food retailers, farmers markets, and breastfeeding among US municipalities, 2014–2021: National Survey of Community-Based Policy and Environmental Supports for Healthy Eating and Active Living (CBS-HEAL). Prev. Chronic Dis..

[19] U.S. Census Bureau (2021). 2017 Census of Governments. https://www.census.gov/data/tables/2017/econ/gus/2017-governments.html.

[20] U.S. Census Bureau (2022). American Community Survey (ACS). https://www.census.gov/programs-surveys/acs.

[21] U.S. Census Bureau (2021). Geographic Levels. https://www.census.gov/programs-surveys/economic-census/guidance-geographies/levels.html.

[22] U.S. Department of Agriculture ERS (2022). Rural Poverty and Well-Being. https://www.ers.usda.gov/topics/rural-economy-population/rural-poverty-well-being/.

[23] CityHealth (2024). Healthy Food Purchasing. https://www.cityhealth.org/our-policy-package/healthy-food-purchasing/.

[24] Lange S.J., Calancie L., Onufrak S.J., Reddy K.T., Palmer A., Lowry Warnock A. (2021). Associations between food policy councils and policies that support healthy food access: A national survey of community policy supports. Nutrients.

[25] Oza-Frank R., Warnock A.L., Calancie L., Bassarab K., Palmer A., Cooksey Stowers K., Harris D. (2025). Food policy councils and healthy food access policies: A 2021 national survey of community policy supports. Prev. Chronic Dis..

[26] Coplen A.K., Cuneo M. (2015). Dissolved: Lessons learned from the Portland Multnomah food policy council. J. Agric. Food Syst. Community Dev..

[27] Schiff R., Levkoe C.Z., Wilkinson A. (2022). Food policy councils: A 20-Year scoping review (1999–2019). Front. Sustain. Food Syst..

[28] Wood M., Robles B., Beltran J., Kuo T. (2024). Integrating healthy nutrition standards and practices into food service contracting in a large US county government. Prev. Chronic Dis..

[29] Cradock A.L., Kenney E.L., McHugh A., Conley L., Mozaffarian R.S., Reiner J.F., Gortmaker S.L. (2015). Evaluating the impact of the healthy beverage executive order for city agencies in Boston, Massachusetts, 2011–2013. Prev. Chronic Dis..

[30] Mason M., Zaganjor H., Bozlak C.T., Lammel-Harmon C., Gomez-Feliciano L., Becker A.B. (2014). Working with community partners to implement and evaluate the Chicago Park District’s 100% Healthier Snack Vending Initiative. Prev. Chronic Dis..

[31] Crombie A.P., Funderburk L.K., Smith T.J., McGraw S.M., Walker L.A., Champagne C.M., Allen H.R., Margolis L.M., McClung H.L., Young A.J. (2013). Effects of modified foodservice practices in military dining facilities on ad libitum nutritional intake of US army soldiers. J. Acad. Nutr. Diet..

[32] Cullen K.W., Watson K., Zakeri I. (2008). Improvements in middle school student dietary intake after implementation of the Texas Public School Nutrition Policy. Am. J. Public Health.

[33] Eneli I.U., Oza-Frank R., Grover K., Miller R., Kelleher K. (2014). Instituting a sugar-sweetened beverage ban: Experience from a children’s hospital. Am. J. Public Health.

[34] Narain K., Mata A., Flores J. (2016). Nutrition policy decreases sugar-sweetened beverages in municipal parks: Lessons learned from Carson, California. J. Public Health Manag. Pract..

[35] French S.A. (2003). Pricing effects on food choices. J. Nutr..

[36] Moore L.V., Carlson S.A., Onufrak S., Carroll D.D., Galuska D. (2017). Development and implementation of a local government survey to measure community supports for healthy eating and active living. Prev. Med. Rep..

[37] Oza-Frank R., McCarthy C., Harris D.M., Blanck H.M., White-Cooper S., Lowry Warnock A. (2026). Assessment of the Presence, Content, and Gaps in Municipal Food Service Guidelines: An Analysis of Policies in the 20 Most Populous U.S. Cities, 2017–2023. Am. J. Health Promot..

